# 3D Bioprinted Human Skeletal Muscle Constructs for Muscle Function Restoration

**DOI:** 10.1038/s41598-018-29968-5

**Published:** 2018-08-17

**Authors:** Ji Hyun Kim, Young-Joon Seol, In Kap Ko, Hyun-Wook Kang, Young Koo Lee, James J. Yoo, Anthony Atala, Sang Jin Lee

**Affiliations:** 1 Wake Forest Institute for Regenerative Medicine, Wake Forest School of Medicine, Winston-Salem, NC 27157 United States; 2 School of Biomedical Engineering and Sciences, Wake Forest University-Virginia Tech, Winston-Salem, NC 27157 United States; 3 Department of Orthopedic Surgery, Soonchunhyang University Bucheon Hospital, Bucheon, Gyeonggi-Do 420-726 Republic of Korea

## Abstract

A bioengineered skeletal muscle tissue as an alternative for autologous tissue flaps, which mimics the structural and functional characteristics of the native tissue, is needed for reconstructive surgery. Rapid progress in the cell-based tissue engineering principle has enabled *in vitro* creation of cellularized muscle-like constructs; however, the current fabrication methods are still limited to build a three-dimensional (3D) muscle construct with a highly viable, organized cellular structure with the potential for a future human trial. Here, we applied 3D bioprinting strategy to fabricate an implantable, bioengineered skeletal muscle tissue composed of human primary muscle progenitor cells (hMPCs). The bioprinted skeletal muscle tissue showed a highly organized multi-layered muscle bundle made by viable, densely packed, and aligned myofiber-like structures. Our *in vivo* study presented that the bioprinted muscle constructs reached 82% of functional recovery in a rodent model of tibialis anterior (TA) muscle defect at 8 weeks of post-implantation. In addition, histological and immunohistological examinations indicated that the bioprinted muscle constructs were well integrated with host vascular and neural networks. We demonstrated the potential of the use of the 3D bioprinted skeletal muscle with a spatially organized structure that can reconstruct the extensive muscle defects.

## Introduction

Skeletal muscle injuries due to trauma or tumor ablation usually require a reconstructive procedure to restore normal tissue function. In the United States alone, approximately 4.5 million patients undergo reconstructive surgeries annually^1^. In many cases, extensive muscle defect results in functional impairment with severe physical deformity^2,3^. The standard of care is an autologous muscle pedicle flap from adjacent regions; however, host muscle tissue availability and donor site morbidity may make this strategy challenging^4^. Recent advances in cell therapy provide alternatives to regenerate muscle tissue for functional augmentation^5^. Injection of cultured cells has shown some efficacy^6–8^; however, this approach can be unrealistic to treat the muscle defect due to low cell engraftment and survival of the injected cells^9,10^. Therefore, bioengineering of an implantable muscle construct that can restore the normal muscle function is an attractive possibility^9,11,12^.

In recent decades, researchers have focused on mimicking the ultrastructure of native muscle tissue that is composed of highly oriented myofibers. The structural organization of skeletal muscle with multiple myofiber bundles is vital for the muscle contraction and functionality^13,14^. Controlling organization of bioengineered muscle tissue *in vitro* should be essential for functional tissue restoration after implantation *in vivo*^15^. Thus, the ability to recapitulate the organization and function of the native skeletal muscle is the most important element in bioengineered skeletal muscle tissues^16^. To build the muscular organization using the alignment of single cells in the bioengineered skeletal muscle constructs *in vitro*, several attempts have been reported. Due to recent advancement in microfabrication technologies, the cellular orientation of muscle cells has been controlled via microtopographic cues^17–24^. The mechanical stimulation^25,26^ and electrical fields^21,27^ have also been tested to align the muscle cells in biomaterial scaffolds. These strategies could pre-align the muscle cells and improve their functionality *in vitro*; however, they only allowed micron-scale tissue or single-layered muscle bundle constructions that may be not suitable for treating extensive muscle defect^10,28–32^.

Recent advances in 3D bioprinting technologies enable to bioengineer various functional tissue constructs with complex geometry by building up cell-laden hydrogels in a layer-by-layer fashion^33–35^. We have previously developed a novel bioprinting method, integrated tissue-organ printing (ITOP) system that can generate a 3D freeform shape with multiple cell types and biomaterials, resulting in the fabrication of various human-scale tissue architectures for translational applications^36^. In our previous work, we utilized this ITOP system to create an organized muscle construct 15 × 5 × 1 mm^3^ in dimension containing mouse myoblast cell line, which composed of pre-aligned muscle fibers within multi-layered myofiber bundles. The outcomes showed that the bioprinted organized muscle constructs could mature into functional muscle *in vivo* when implanted subcutaneously in rats.

Based on this initial success, we investigated the feasibility of using 3D bioprinted muscle constructs for treating extensive skeletal muscle defects. In this study, we created skeletal muscle constructs (mm^3^–cm^3^ scale) with the structural integrity and skeletal muscle tissue organization for functional muscle tissue reconstruction. Also, muscle progenitor cells (MPCs) used in this study were isolated from human muscle tissue biopsies for further clinical relevance. Evaluations for the muscle characteristics *in vitro* were performed. Muscle tissue regeneration and functional recovery were evaluated using a rodent muscle defect model of 30–40% of tibialis anterior (TA) muscle loss with ablation of extensor digitorum longus (EDL) and extensor hallucis longus (EHL) muscles^10^ to determine the feasibility to treat critical-sized skeletal muscle injuries.

## Results

### 3D bioprinted muscle constructs with structural mimicry *in vitro*

**Figure 1:**
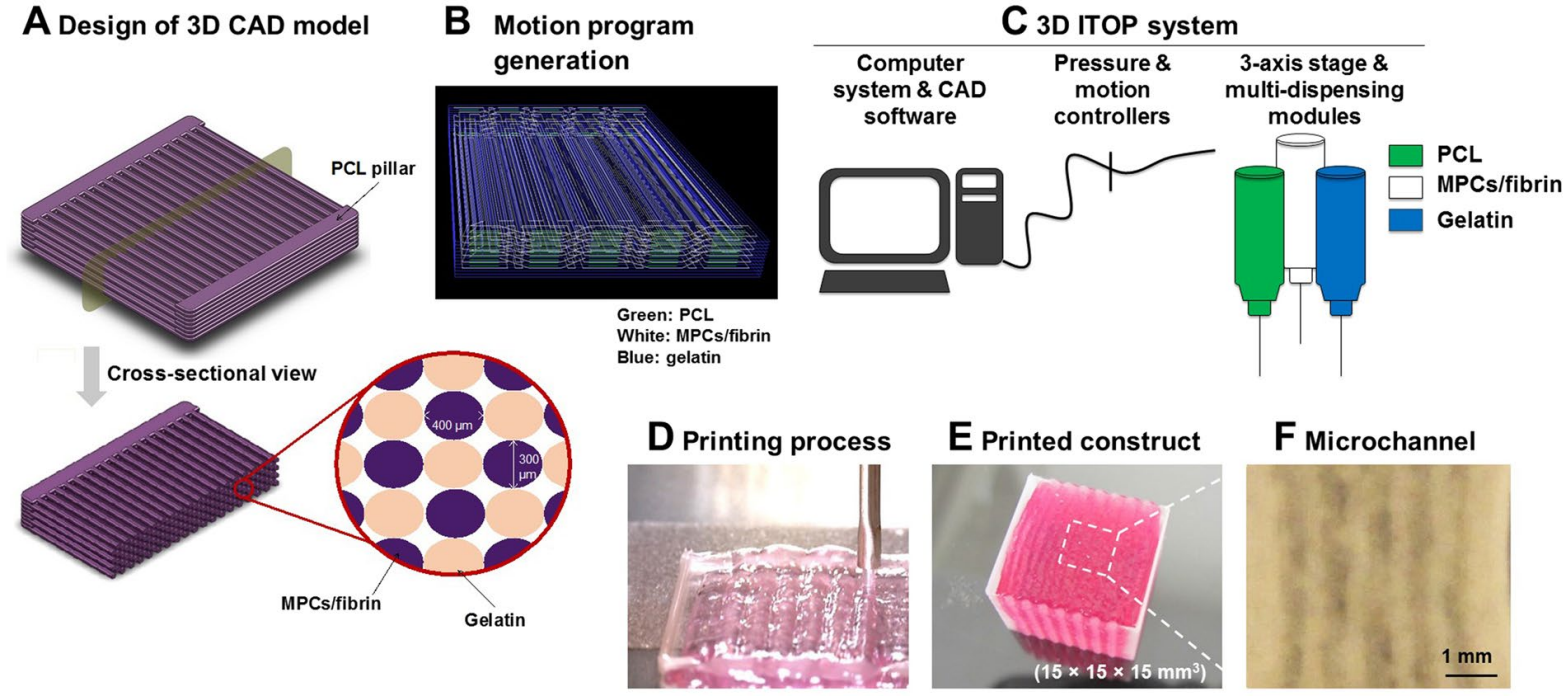
Bioprinting of skeletal muscle. (**A**) Design concept using 3D CAD modeling and (**B**) motion program generation of the bioprinted muscle construct. The code includes XYX stage movement and actuating pneumatic pressure. (**C,D**) Bioprinting process using ITOP system. (**C**) The motion program was transferred to the operating computer of ITOP. The cell-laden bioink containing hMPCs, the acellular sacrificing hydrogel, and the supporting PCL pillar were loaded in the multi-dispensing modules. (**D**) All three components were printed in a layer-by-layer fashion. (**E**) The bioprinted skeletal muscle constructs composed of multi-layered myofiber bundles were fabricated up to 15 × 15 × 15 mm^3^ in dimension. The thickness of the printed muscle construct was determined by controlling the number of stacking myofiber bundles. (**F**) Microchannels in the constructs created after the removal of the sacrificial patterns to maintain the viability of printed cells.

A bioengineered skeletal muscle construct with the ultrastructural organization similar to the native muscle was designed and fabricated by the ITOP technology (Fig. 1A,B). This muscle construct consisted of three components: (i) a human muscle progenitor cell (hMPC)-laden hydrogel bioink, (ii) a sacrificing acellular gelatin hydrogel bioink, and (iii) a supporting poly(ε-caprolactone) (PCL) polymer (Fig. 1C). To create the cellular organization in the printed skeletal muscle structure, multiple strips of the cell-laden bioinks were patterned in parallel to one another, which were anchored to the PCL pillar structure (Fig. 1D). The thickness of the printed constructs was determined and controlled by the number of stacking layers of cell-laden bioinks. A printed PCL pillar structure allowed to align printed cells longitudinally in response to mechanical cues and to maintain the structural integrity of multi-layered muscle constructs after printing. To maintain the viability of printed cells within large-scale muscle constructs (up to 15 × 15 × 15 mm^3^, Fig. 1E), we created microchannels between the cell-laden patterns based on the diffusion limit of oxygen and nutrients, ~200 µm^10,32^ (Fig. 1F).

We evaluated the bioprinted skeletal muscle constructs containing 3 × 10^6^ cells/ml of hMPCs *in vitro* on the viability, differentiation capacity to form multinucleated myofibers, and the cellular orientation in the printed constructs. The printed constructs were cultured for 1 day in growth medium and then induced differentiation for 9 days in differentiation medium. In the live/dead analysis, the bioprinted muscle constructs had highly organized multiple myofiber bundles in which hMPCs were longitudinally aligned along the printed pattern direction (Supplementary Fig. 1A). Microchannels between the bundles of myofibers were also observed. The maturation of the bioprinted muscle was confirmed by myosin heavy chain (MHC) immunostaining (Supplementary Fig. 1B).

**Figure 2:**
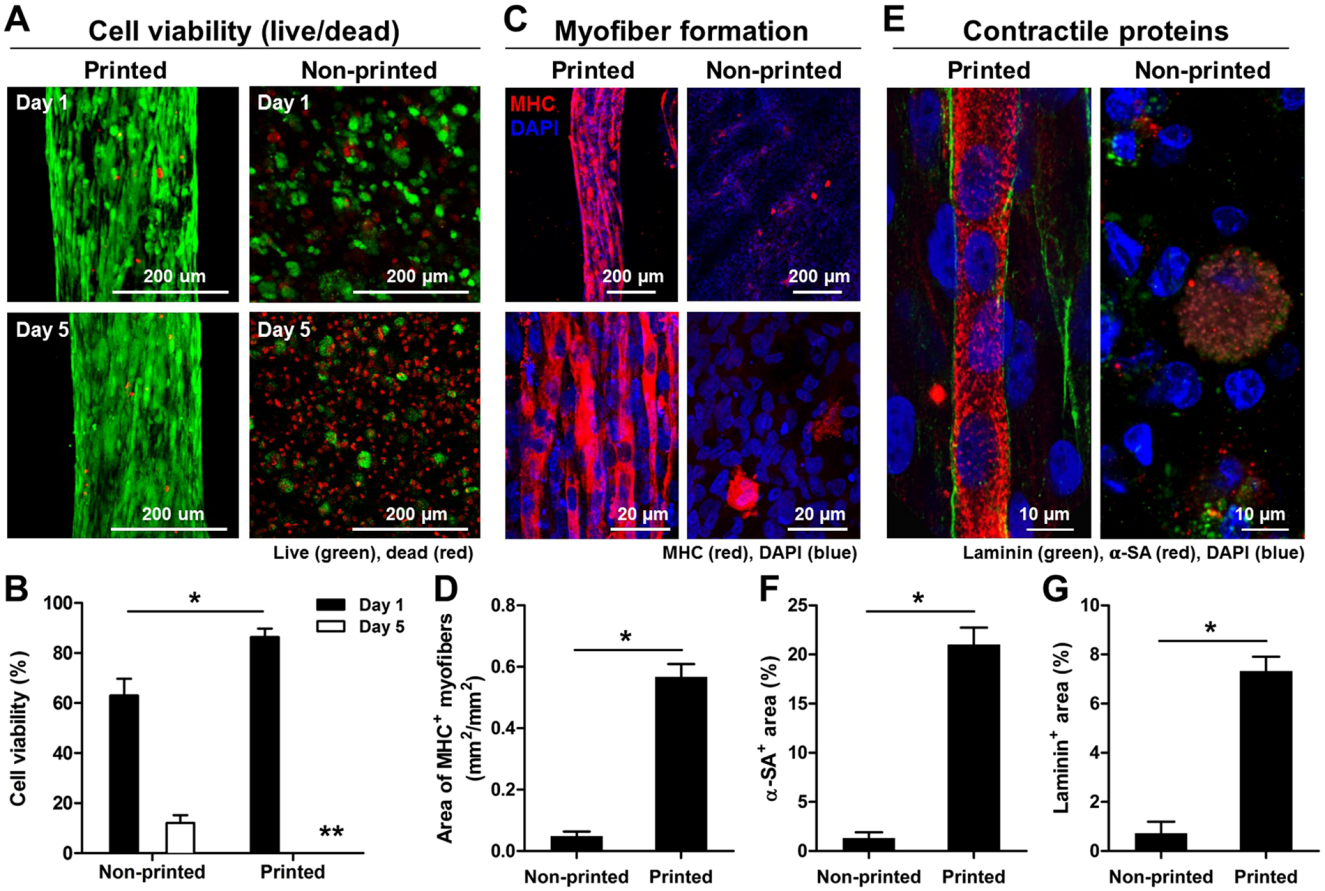
*In vitro* evaluations of bioprinted muscle constructs compared with non-printed constructs. (**A**) Representative Live/Dead staining images and (**B**) cell viability (%) at 1 and 5 days in culture (n = 4, 4 random fields/sample, *P < 0.05, **not measurable because of too confluence - % viability was over 90%). (**C**) Immunofluorescent staining for MHC after 7 day-differentiation and (**D**) quantification of area of MHC + myofibers (*n* = 3, 4–7 random images/sample, *P < 0.05). Human MPCs in the construct showed enhanced myofiber formation with unidirectional cell alignment. (**E**) Double-immunostaining for α-SA (red)/laminin (green) indicates the presence of cross-striated myofibers surrounded by laminin matrix in the printed construct. Quantification of (**F**) α-SA^+^ area (%) and (**G**) laminin^+^ area (%) (n = 3, *P < 0.05).

To determine the importance of organized architecture and microchannel structure on skeletal muscle construction, the bioprinted and non-printed (hMPCs in hydrogel without printing) constructs were prepared with the same cell density (30 × 10^6^ cells/ml) and dimension (10 × 10 × 3 mm^3^), and the cell viability and differentiation were measured during *in vitro* culture. In the live/dead assay staining, the bioprinted muscle constructs showed high cell viability (86.4 ± 3.5%) compared to the non-printed muscle constructs (63.0 ± 6.7%) at 1 day in culture; however, most of the cells in the non-printed constructs died at 5 days, while high cell viability was maintained in the printing constructs (Fig. 2A,B; *n* = 4 and 4 random fields/sample, Student *t*-test, *P < 0.05). MHC^+^ myofibers in the bioprinted constructs showed an 11.53-fold increase when compared with the non-printed constructs at 7 days of differentiation, and the myofibers were densely packed and aligned (Fig. 2C,D; *n* = 3 and 4–7 random fields/sample, Student *t*-test, *P < 0.05). In the bioprinted constructs, cross-striated myofibers surrounded by basal lamina-like matrices were observed in double-immunostaining for α-sarcomeric actin (α-SA) and laminin, indicating the muscle contractile properties (Fig. 2E–G, *n* = 3 and 4–7 random fields/sample, Student *t*-test, *P < 0.05). These results show that the 3D printed organized muscle structure can accelerate the tissue maturation, while the microchannel structure can allow the diffusion of nutrient and oxygen to maintain the cell viability in the printed constructs.

### Effect of initial cell density in bioprinted muscle construct

**Figure 3:**
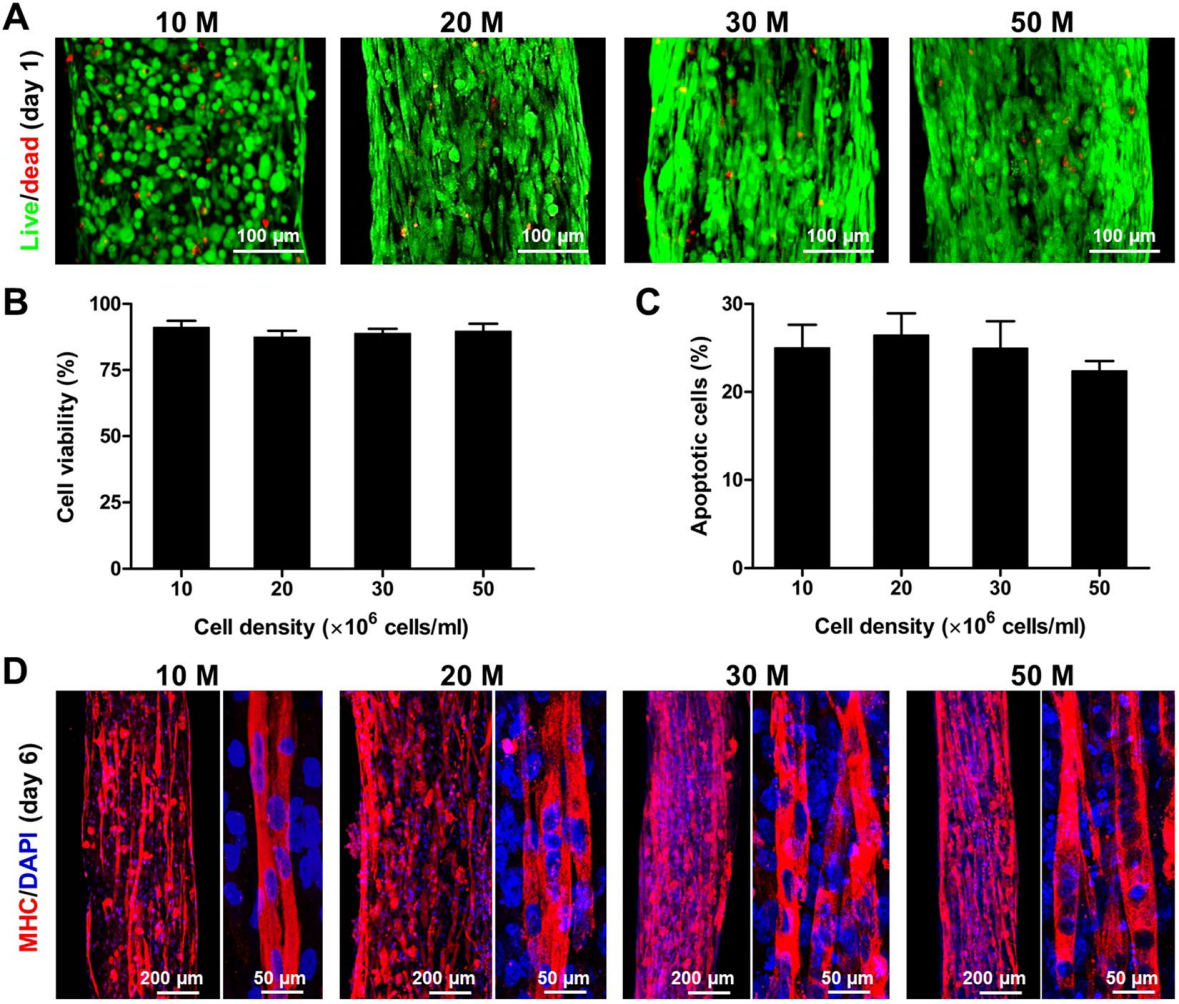
*In vitro* cell density optimization. (**A**) Live/dead staining images and (**B**) quantification of bioprinted muscle constructs with cell densities of 10, 20, 30, and 50 × 10^6^ cells/ml at 1 day in culture (*n* = 6, 5 random fields/sample, no significant difference). (**C**) TUNEL assay of bioprinted muscle constructs after 6 days in culture. Apoptotic cells were calculated with different cell densities (*n* = 3, 5 random fields/sample, no significant difference). (**D**) MHC immunofluorescent images of bioprinted muscle constructs at 6 days in culture (after 5-day differentiation). Representative immunofluorescent images for MHC (red) showed that bioprinted hMPCs in the constructs with different cell densities were formed into longitudinally aligned myofibers.

We printed the organized muscle constructs with various cell densities (10, 20, 30, and 50 × 10^6^ cells/ml) and evaluated the cell viability and differentiation *in vitro*. Approximately 90% live cells were determined at 1 day in all constructs with no significant differences (Fig. 3A,B; *n* = 6 and 5 random fields/sample, ANOVA and *post hoc* Tukey test), and approx. 25% apoptotic cells were detected at 6 days in culture with no significant differences among groups (Fig. 3C; *n* = 3 and 5 random fields/sample, ANOVA and *post hoc* Tukey test) as confirmed by TUNEL staining assay. The differentiated myofibers were strongly expressed MHC in all groups with cells aligned longitudinally in the bioprinted constructs at 6 days in culture (Fig. 3D). The density of MHC^+^ myofibers tended to increase with increasing cell density in the bioprinted muscle constructs. These results indicate that the ITOP system could build the skeletal muscle constructs with highly viable and differentiated into highly aligned, densely packed myofibers over a broad range of cell densities.

**Figure 4:**
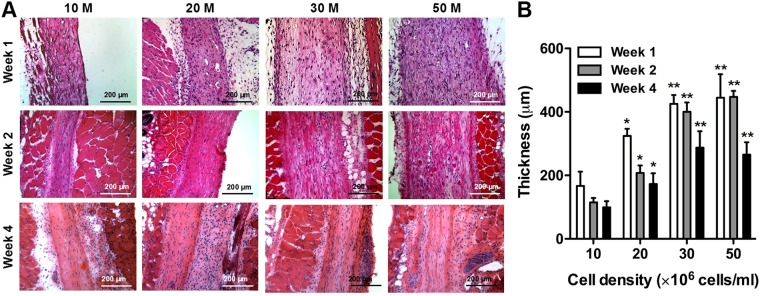
*In vivo* cell density optimization on dimensional maintenance. (**A**) H&E-stained images of longitudinal cross-sections of bioprinted muscle constructs with different cell densities at 1, 2 and 4 weeks after implantation. (**B**) The thickness of bioprinted muscle constructs as measured by H&E-stained sections (*n* = 4, 3 random regions/sample). Thickness of the constructs increased with cell density, but there was no significant difference between 30 and 50 × 10^6^ cells/ml at 2 weeks and 4 weeks (*P < 0.05 compared with 10 × 10^6^ cells/ml, **P < 0.05 with 10 × 10^6^ cells/ml and 20 × 10^6^ cells/ml).

To determine the optimal cell density in the bioprinted muscle constructs in terms of the dimensional maintenance *in vivo*, the bioprinted muscle constructs (10 × 10 × 3 mm^3^) with different cell densities (10, 20, 30, and 50 × 10^6^ cells/ml of hMPCs) were implanted subcutaneously in athymic mice (total 48 nu/nu mice, male, 6–8 weeks old). Hematoxylin and eosin (H&E) stained images showed that the bioprinted muscle constructs of multi-layered, longitudinally aligned patterns were maintained until 4 weeks after implantation (Fig. 4A). The thickness of the implanted constructs (*n* = 4 and 3 random fields/sample) increased with cell density, but there was no significant difference between 30 × 10^6^ cells/ml and 50 × 10^6^ cells/ml groups at 1, 2, and 4 weeks (Fig. 4B; ANOVA and Tukey test, *P *<* 0.05 compared with 10 × 10^6^ cells/ml, **P *<* 0.05 compared with 10 × 10^6^ cells/ml and 20 × 10^6^ cells/ml). In the 30 × 10^6^ cells/ml and 50 × 10^6^ cells/ml groups, the thickness of the construct was maintained until 2 weeks and decreased at 4 weeks.

**Figure 5:**
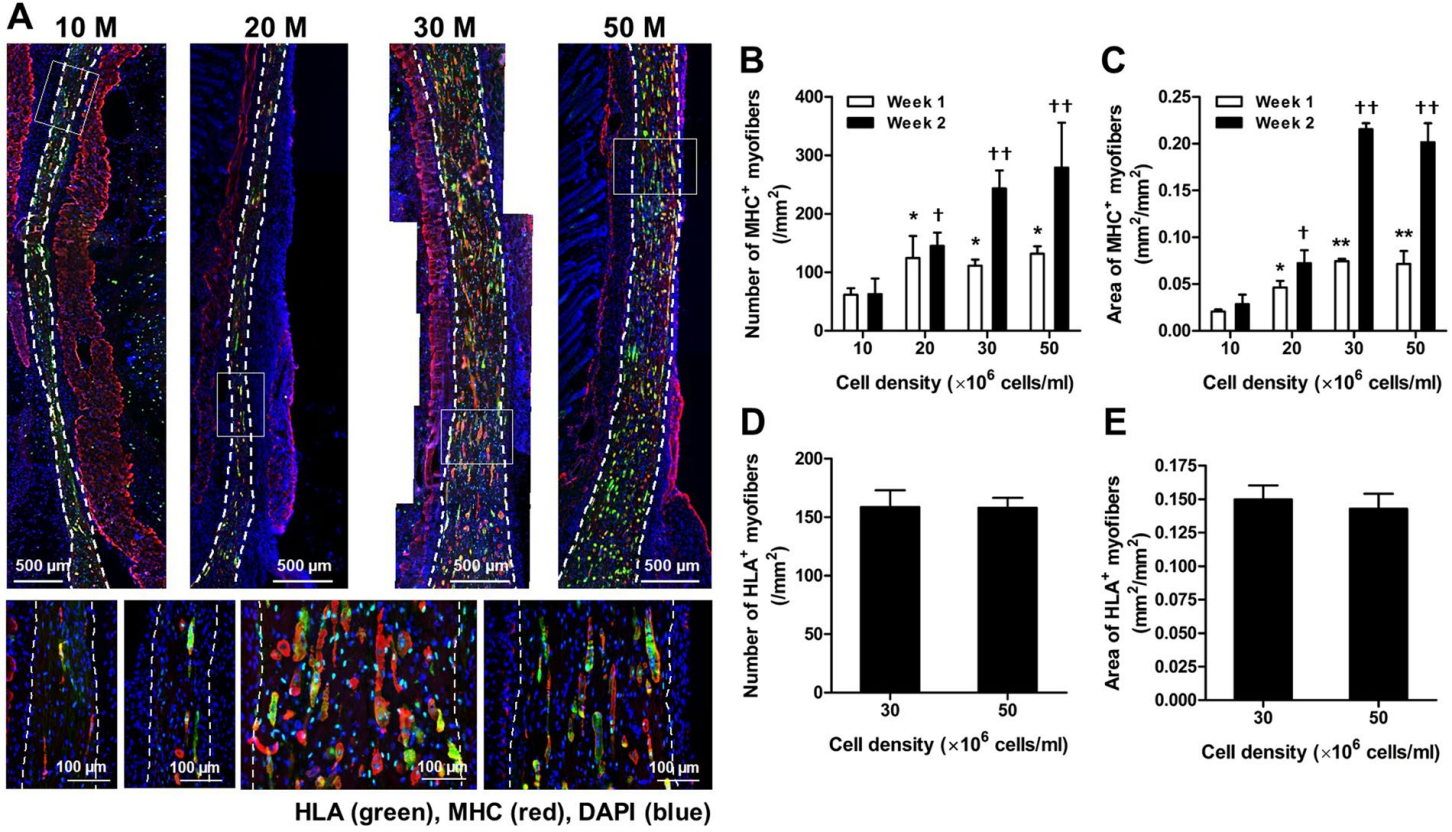
Ectopic skeletal muscle regeneration. (**A**) Representative immunofluorescent images for MHC (red)/HLA (green) at 2 weeks after implantation. Double-immunostained MHC^+^/HLA^+^ myofibers in bioprinted constructs indicate newly formed skeletal muscle. (**B**) Numbers of MHC^+^ myofibers and (**C**) areas of MHC^+^ myofibers (*n* = 4, 3 random regions/sample). There is an increasing trend of skeletal muscle tissue formation with increasing cell density, but no significant difference between 30 and 50 × 10^6^ cells/ml at 1 week and 2 weeks after implantation (*P *<* 0.05 compared with 10 × 10^6^ cells/ml at 1 week, **P *<* 0.05 compared with 10 and 20 × 10^6^ cells/ml at 1 week, ^†^*P* *<* 0.05 compared with 10 × 10^6^ cells/ml at 2 weeks, and ^††^*P* *<* 0.05 compared with 10 and 20 × 10^6^ cells/ml at 2 weeks). (**D**) Numbers of HLA + myofibers and (**E**) areas of HLA^+^ myofibers (*n* = 4, 3 random regions/sample). There is no significant difference between 30 × 10^6^ cells/ml and 50 × 10^6^ cells/ml at 2 weeks after implantation.

The retrieved muscle constructs were also characterized using double-immunostaining of MHC and human leukocyte antigen A (HLA) antibodies. MHC^+^ myofibers were identified in all implanted bioprinted muscle constructs at 2 weeks, and they were aligned longitudinally along the implants (Fig. 5A). Newly formed myofibers in the implanted constructs were measured as the number of MHC^+^ myofibers (/mm^2^) and the area of MHC^+^ myofibers (mm^2^/mm^2^) (Fig. 5B,C; *n* *=* 4 and 3 random fields/sample). These parameters were increased with increasing the cell density in the implanted constructs, but there was not significantly different between 30 × 10^6^ cells/ml and 50 × 10^6^ cells/ml groups at 1 and 2 weeks after implantation (ANOVA and Tukey test, *P *<* 0.05 compared with 10 × 10^6^ cells/ml at 1 week, **P *<* 0.05 compared with 10 and 20 × 10^6^ cells/ml at 1 week, ^†^*P* *<* 0.05 compared with 10 × 10^6^ cells/ml at 2 weeks, and ^††^*P* *<* 0.05 compared with 10 and 20 × 10^6^ cells/ml at 2 weeks). MHC^+^/HLA^+^ double-immunostained images determined that newly formed myofibers were derived from the implanted bioprinted muscle constructs (Fig. 5A). There was no significant difference in a number of HLA^+^ myofibers (/mm^2^) and area of HLA^+^ myofibers (/mm^2^) between 30 and 50 × 10^6^ cells/ml at 2 weeks (Fig. 5D,E; Student *t*-test). In addition, over 80% MHC^+^ myofibers in all groups were expressed by HLA^+^ at 2 weeks of implantation. Vascularization and neural integration were also demonstrated with von Willebrand factor (vWF) and neurofilament (NF)/alpha-Bungarotoxin (α-Btx) double-immunostaining at 2 weeks, respectively (Supplementary Fig. 2). The vWF^+^ vessels and NF^+^ nerves were observed throughout the implants and in the surrounding tissues (white arrows). Results indicate that the optimal cell density of the bioprinted muscle constructs is 30 × 10^6^ cells/ml.

### Structural and functional restoration by implantation of bioprinted muscle constructs

**Figure 6:**
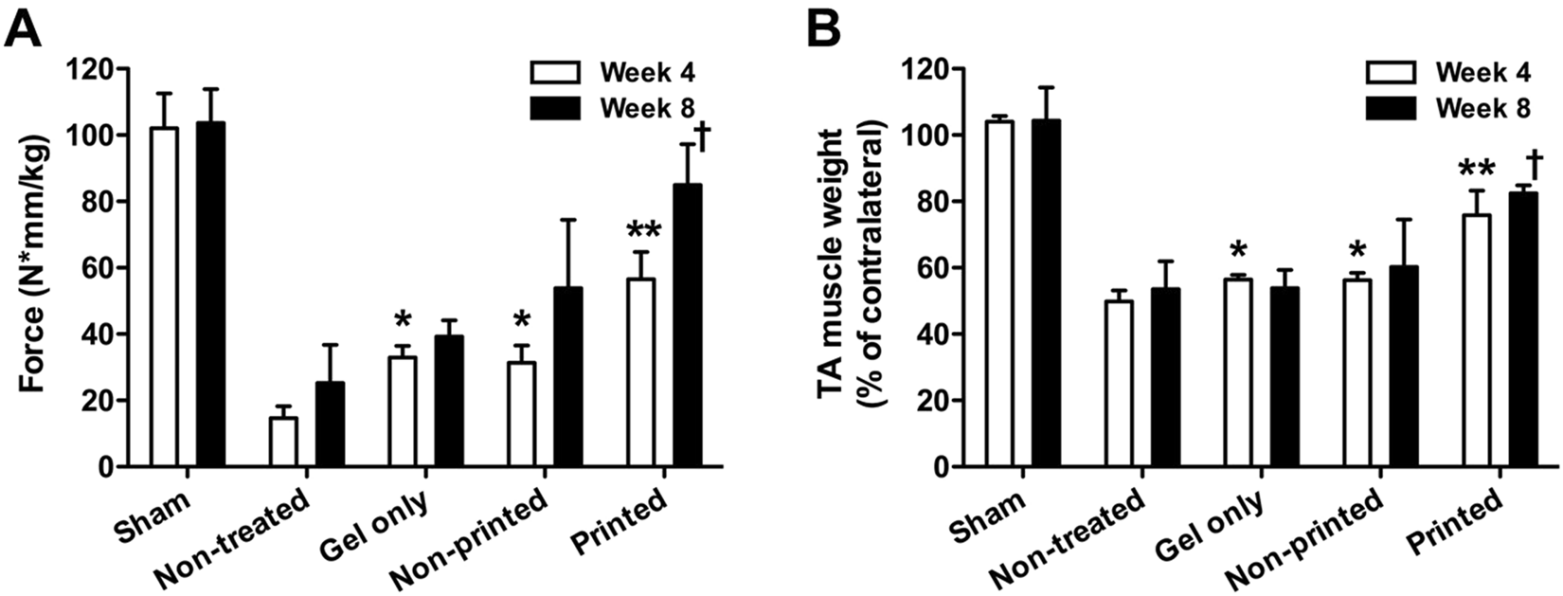
Rat TA muscle defect model. (**A**) Tetanic force (N · mm/kg) and (**B**) TA muscle weight (% of contralateral normal TA muscle) were measured at 4 and 8 weeks after implantation (n = 3 per group, triple measures per sample). Tetanic force and muscle weight of bioprinted muscle constructs-implanted group had significantly increased when compared with other groups (*P < 0.05 compared with non-treated group at 4 weeks, **P < 0.05 compared with non-treated, gel only, and non-printed groups at 4 weeks, and ^†^*P* < 0.05 compared with non-treated, gel only, and non-printed groups at 8 weeks).

Based on the *in vitro* and *in vivo* results, we next investigated the feasibility of the bioprinted muscle constructs to restore the muscle function using the rat TA muscle defect model. A muscle defect was created by excision of 30–40% of original TA muscle mass following ablation of EDL and EHL muscles in nude rats (Supplementary Fig. 3A)^10^. This TA muscle defect causes irreversible anatomic and functional deficits for 6 months post-injury without any treatment^37^. The bioprinted muscle constructs were implanted into the defect region in TA muscle, and sham control (without defect), non-treated (defect only), gel only (non-printed and no cells), and non-printed (random structure with cells) constructs were used for comparison (30 RNU rats, *n* = 3, One-way ANOVA and Turkey test to compare group differences in each time point and Student *t*-test to compare time differences in each group). The *in vivo* functional analysis was performed by measuring tetanic muscle force of the muscle with peroneal nerve stimulation. We used the bioprinted muscle constructs with an initial cell density of 30 × 10^6^ cells/ml. After *in vitro* culture, the bioprinted muscle constructs were implanted in the defect site following removal of the PCL frame to fit the defect region in TA muscle (10 × 7 × 3 mm^3^). In the gross examination of the harvested TA muscles, the surgically created defect resulted in severe muscular atrophy in the non-treated, gel only, and non-printed groups at 4 and 8 weeks post-injury, while the bioprinted group maintained their original muscle volume during the 8-week period (Supplementary Fig. 3B). More importantly, at 4 and 8 weeks after implantation, only the bioprinted muscle group showed a significant increase of the tetanic muscle force and TA muscle weight when compared with other groups (Fig. 6; ANOVA and Turkey test, **P < 0.05 at 4 weeks and ^†^*P* < 0.05 at 8 weeks). However, no significant differences in the tetanic muscle force and TA weight of the gel-only and non-printed groups were determined when compared with the non-treated group at 8 weeks after implantation. By 8 weeks, the muscle force in the bioprinted group reached to 85.0 ± 12.3 N*mm/kg that was 82% restoration of TA muscle force compared with normal TA muscle. TA muscle weight in bioprinted group increased 1.52 and 1.54-fold compared to that in the non-printed group at 4 and 8 weeks, respectively. By 8 weeks, muscle weight had recovered up to 82.5 ± 2.4% of the contralateral normal TA muscle.

### Newly formed myofibers and tissue maturation by implantation of bioprinted muscle constructs

**Figure 7:**
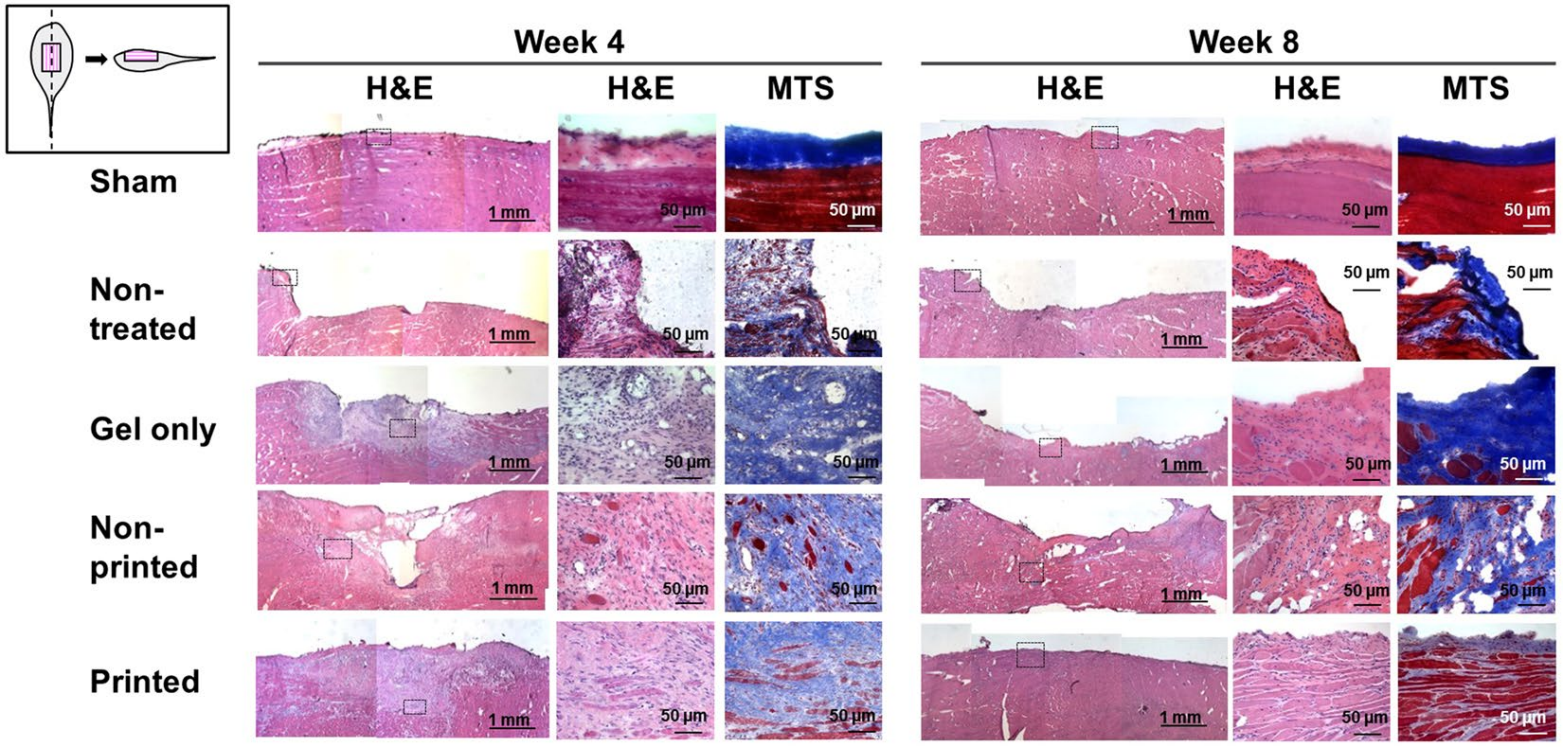
Histological examinations. Histological images showed highly aligned newly formed myofibers in bioprinted constructs with superior muscle volume maintenance at 4 and 8 weeks post-implantation, while severe muscle atrophy and limited muscle regeneration were determined in other groups. Squares in left column indicate areas shown in detail with high magnifications. MTS, Masson’s trichrome staining.

Improved structural outcomes by implantation of the bioprinted muscle constructs were examined by histological and immunofluorescent analyses. The analyses were performed with longitudinal sections of the TA muscles to examine overall implanted regions and the alignment along with native muscle orientation. In H&E and Masson’s trichrome staining, the bioprinted muscle group showed superior muscle volume maintenance and myofiber formation with organized architecture, while the other groups showed limited muscle volume recovery and tissue development (Fig. 7 and Supplementary Fig. 4).

**Figure 8:**
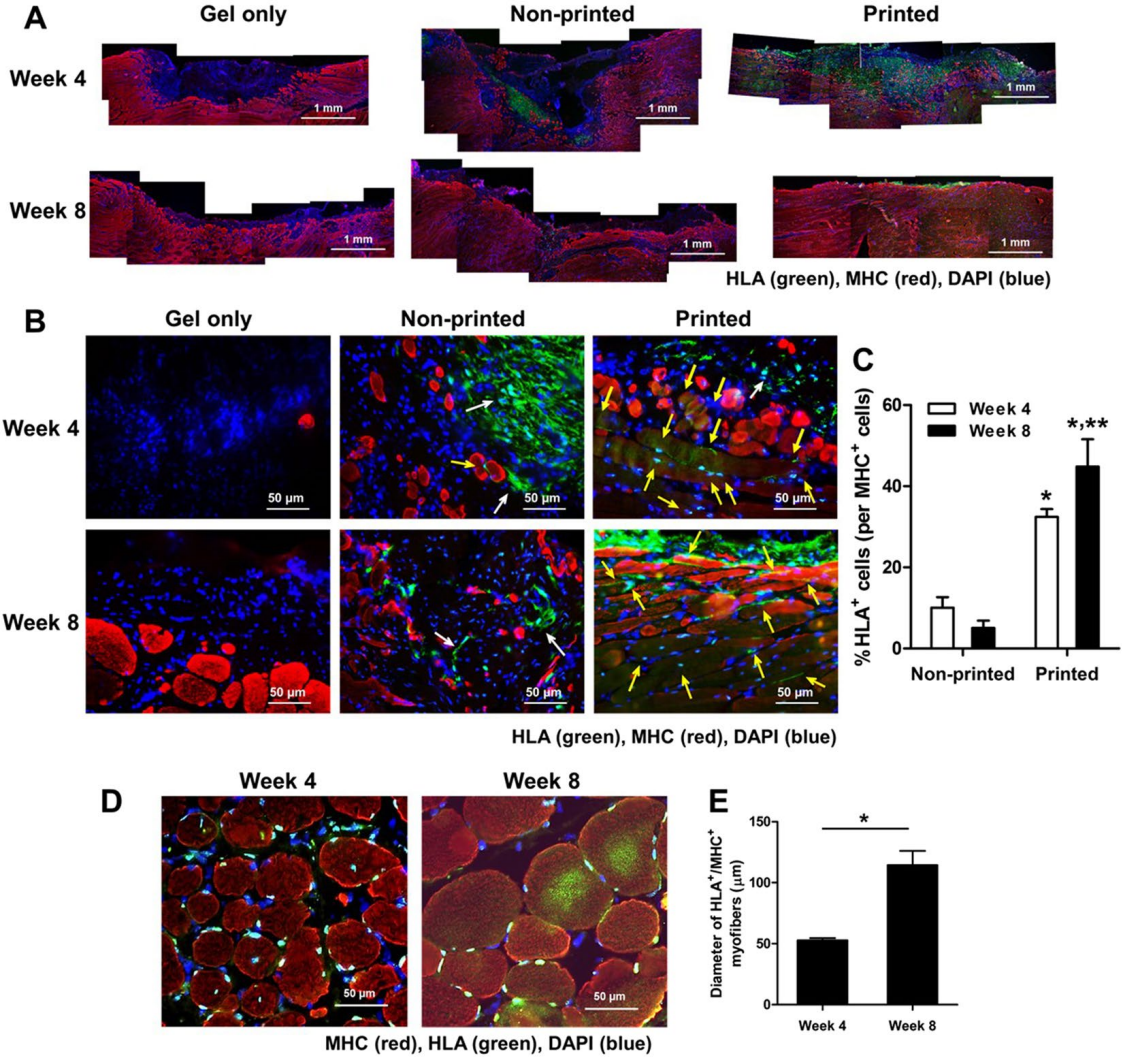
Immunofluorescent analysis of bioprinted muscle constructs on newly formed muscle. (**A**) Double-immunostaining of MHC/HLA of the retrieved TA muscles (MHC: red, HLA: green). (**B**) Double-immunostaining of MHC/HLA of the implanted regions. (**C**) Quantification of co-localized HLA^+^/MHC^+^ cells (% of HLA^+^ cells per MHC^+^ cells (*n* = 3, *P < 0.05 compared with non-printed group and **P < 0.05 between 4 and 8 weeks). Higher skeletal muscle regeneration in bioprinted construct was observed at 4 and 8 weeks of implantation. MHC^+^/HLA^+^ newly formed myofibers in bioprinted constructs indicate that the implanted hMPCs contributed to skeletal muscle regeneration in the defect region (MHC: red, HLA: green, MHC^−^/HLA^+^ cells: white arrow, MHC^+^/HLA^+^ cells: yellow arrow). (**D**) A cross-sectional view of double-immunostaining of MHC/HLA of the bioprinted muscle constructs and (**E**) Quantification of the diameter of MHC^+^/HLA^+^ myofibers (µm) (*n* = 3, 3 random fields per sample, *P < 0.05).

To identify the newly formed myofibers in the implanted region, we performed double-immunofluorescent staining of the retrieved TA muscles using MHC and HLA antibodies (Fig. 8A,B). Few MHC^+^ myofibers were determined in the gel-only and non-printed groups at 4 weeks after implantation. In the non-printed group containing hMPCs, only a few HLA^+^ cells remained adjacent to the host muscle tissue at 4 and 8 weeks. In contrast, in the bioprinted group, a higher number of HLA^+^ human cells were detected throughout the implanted region at 4 and 8 weeks after implantation (Fig. 8C; *n* = 3, Student *t*-test, *P < 0.05 compared with the non-printed group, **P < 0.05 between 4 and 8 weeks). Notably, the bioprinted muscle constructs became more matured that significantly contributed to the muscle tissue regeneration, as confirmed by MHC^+^/HLA^+^ myofibers (Fig. 8D). The implanted hMPCs were continuously involved in the myofiber formation and maturation with increased myofiber diameter at 8 weeks (Fig. 8E; cross-sectional analysis, *n* = 3, 3 random fields per sample, Student *t*-test, *P < 0.05). Furthermore, most MHC^+^/HLA^+^ myofibers in the TA muscle showed highly aligned and organized architecture. These results indicate that the implanted hMPCs in the bioprinted constructs are viable and matured with maintaining their cellular organization for reconstructing the extensive muscle defect injury.

**Figure 9:**
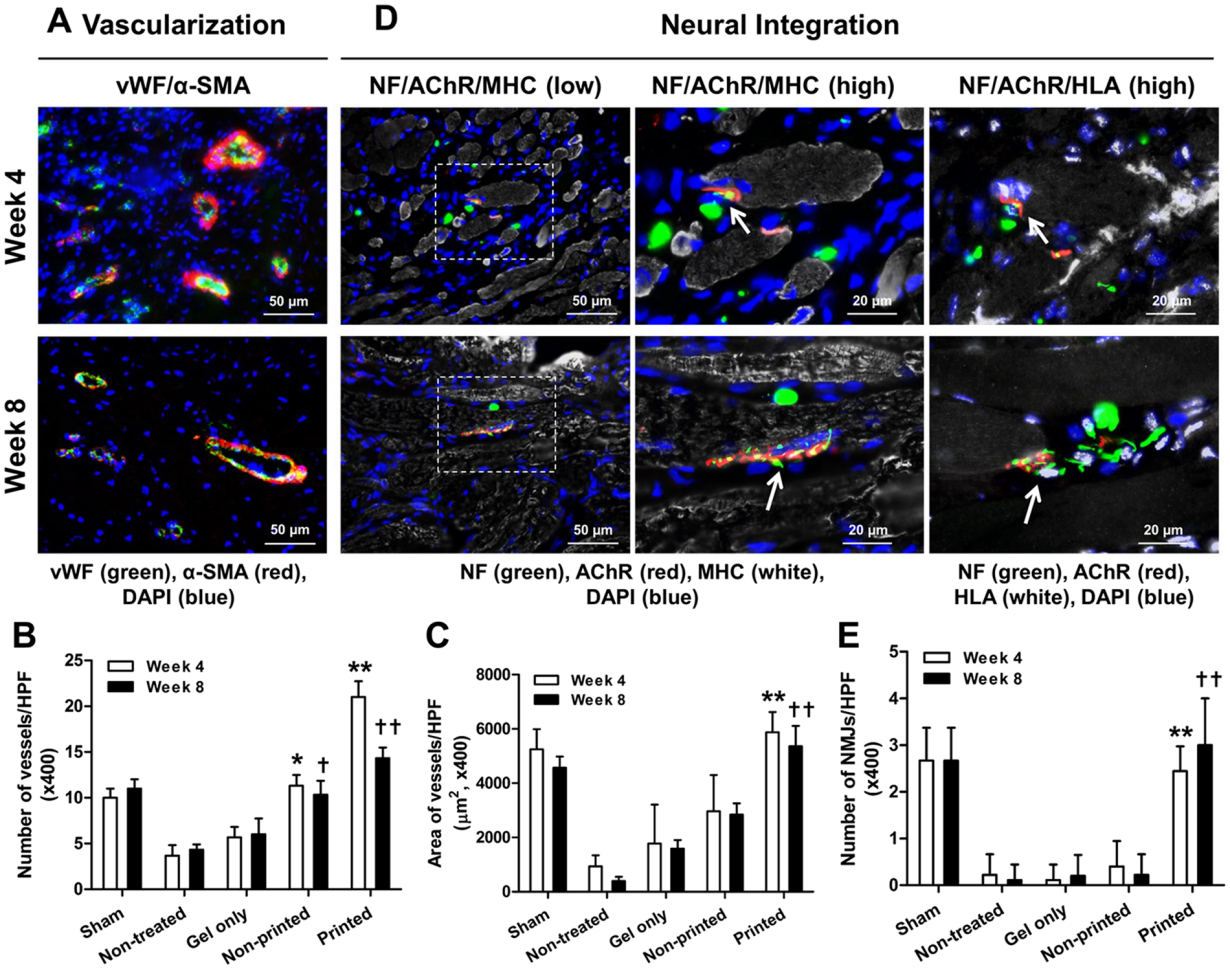
Immunofluorescence of vascularization and neural integration of the implanted bioprinted muscle constructs. (**A**) Immunofluorescent images of vWF (green)/α-SMA (red) of the regenerated TA muscles at 4 and 8 weeks after implantation. Quantification of (**B**) vessels/field and (**C**) area of vessels/field (µm^2^) (*n* = 3, *P < 0.05 compared with non-treated and gel only groups, **P < 0.05 compared with other groups). (**D**) Immunofluorescent images of NF (green)/AChR (red)/MHC (white) and NF (green)/AChR (red)/HLA (white) of the regenerated TA muscles at 4 and 8 weeks after implantation. NF^+^/AChR^+^/MHC^+^ neuromuscular junction (middle column, white arrow) was observed in bioprinted muscle constructs. NF^+^/AChR^+^/HLA^+^ neuromuscular junction (right column) corresponding area NF^+^/AChR^+^/MHC^+^ (middle column) indicates that bioprinted muscle constructs are integrated with host nervous system following implantation. The white arrow indicates neuromuscular junction on hMPC-myofibers. (**E**) Quantification of NMJ/field (×400) (*n* = 3 per group, 3 random fields per sample, *P < 0.05 compared with other groups).

Vascularization and neural integration of the implanted bioprinted muscle constructs were evaluated by double-immunofluorescent staining for vWF/α-smooth muscle actin (α-SMA) and triple-immunofluorescent staining for NF/acetylcholine receptor (AChR)/MHC and NF/AChR/HLA, respectively (Fig. 9 and Supplementary Fig. 5). Immunofluorescent images showed the implanted bioprinted muscle constructs were well-vascularized as confirmed by vWF^+^ and α-SMA^+^ vessels (Fig. 9A–C; *n* = 3, ANOVA and Turkey test, *P < 0.05 compared with non-treated and gel only groups at 4 weeks, **P < 0.05 compared with other groups at 4 weeks, ^†^*P* < 0.05 compared with non-treated and gel only groups at 8 weeks, and ^††^*P* < 0.05 compared with other groups at 8 weeks). Also, host nerve integration was determined by NF^+^ nerve fibers in the implanted region (Fig. 9D). Neuromuscular junction (NMJ) formation, as identified by NF^+^, AChR^+^, and MHC^+^, was clearly determined in the implanted region, and mature NMJs were observed at 8 weeks after implantation (Fig. 9D,E; *n* = 3 per group, 3 random fields per sample, ANOVA and Turkey test, **P < 0.05 compared with other groups at 4 weeks and ^††^*P* < 0.05 compared with other groups at 8 weeks). Immunofluorescent staining for NF/AChR/HLA corresponding to NF/AChR/MHC indicated that the newly formed muscle in the bioprinted constructs could be integrated with host nerve system (Fig. 9D). These outcomes state that the 3D bioprinted muscle constructs can be functional by integration with the host vascular and nerve systems following implantation in the TA muscle defect.

## Discussion

Bioengineering of an implantable muscle construct with structural and functional characteristics of native skeletal muscle is critical for reconstructive surgery. We previously developed the ITOP system that could generate 3D freeform shapes with multiple cell types and biomaterials, resulting in various tissue architectures with biomimetic features^36,38–40^. We also applied this system to fabricate the organized skeletal muscle construct of 15 × 5 × 1 mm^3^ in dimension containing mouse myoblast cell line^36^. To validate the muscle tissue development, this bioprinted tissue structure was eventually implanted subcutaneously in athymic nude rats. The results showed newly formed, oriented myofiber bundles derived from the implanted bioprinted cell-based constructs. In this present study, we extended our bioprinting strategy to repair the muscle defect injuries using the bioprinted skeletal muscle containing human cell source that is more clinically relevant. Unlike other methods^17–20,22^, we were able to build 3D skeletal muscle constructs (up to 15 × 15 × 15 mm^3^) that had long parallel multi-layered bundles of densely packed, highly viable, and aligned myofibers.

The 3D ITOP system used in this study overcame current limitations on both size and spatial organization for the bioengineered skeletal muscle. By simultaneous printing of three components, including the cell-laden hydrogel, the sacrificing acellular hydrogel, and the thermoplastic polymeric pillar structure, we were able to achieve viable skeletal muscle constructs with structural mimicry and cellular function of native skeletal muscle. For the hydrogel-based bioink materials for cell positioning, three major properties are required; (i) relatively higher viscosity to maintain homogenous cell suspension before printing, (ii) strong shear-thinning behavior to reduce the cell damage during printing, and (iii) rapid gelation to build a 3D tissue structure after printing^41^. Our bioink formulation in this study was optimized based on these requirements^36^. In our printing design strategy, the supporting PCL pillar was essential for the cellular alignment, viability, and differentiation^36^. We designed the cell-laden printing beams to be anchored to the PCL pillar that could place the mechanical tension on the cells and drive the cell alignment in a longitudinal direction. The need of the PCL pillar for the cellular alignment and maturation was confirmed by low cell viability in a disorganized structure in the non-printed cellular construct and the printed constructs without PCL pillar^36^. This pillar also supported the structural stability without collapse or deformation during the culture, resulting in maintaining the multi-layered skeletal muscle organization.

A microchannel structure was created in the bioprinted muscle constructs after removal of the sacrificing acellular gelatin hydrogel because the most common drawback of the large-scale cell-based constructs is the limited supply of oxygen and nutrients into the innermost area^31,42^. Limited diffusion capacity in the bioengineered tissue constructs can result in the induction of a necrotic core, which is unexpected for further functional muscle restoration^1,10^. Gelatin is thermo-sensitive, which is liquid above 37 °C and solid below 25 °C. The gelatin-based sacrificing hydrogel was co-printed in parallel with cell-laden patterns at 18 °C. During the printing process, sacrificing gelatin hydrogel patterns supported structural stability the cellular patterns before the crosslinking process. After crosslinking, gelatin, hyaluronic acid (HA), and glycerol were dissolved out during incubation at 37 °C, and the voided space became microchannel in the construct. Our *in vitro* results also showed low cell viability of the large, highly dense constructs (non-printed construct with 30 × 10^6^ cells/ml and 10 × 10 × 3 mm^3^). Failure of non-printed constructs regarding the cell survival and muscle tissue formation emphasizes the need for strategies for efficient oxygen and nutrient supply into the implanted cells in the large-scale tissue constructs. Thus, our bioprinted muscle constructs had the microchannel structure of 300–400 µm in diameter between the printed cellular patterns of myofiber bundles. These microchannels maintained the cell viability in the large-scale skeletal muscle constructs in high cell densities over 30 × 10^6^ cells/ml during *in vitro* culture. Moreover, our results showed that the cellular constructs with microchannels were viable after implantation because the microchannel structure enabled the cells to be alive until the vascular networks were developed in the construct.

To exploit the maximum cellular function, the initial cell density (a range of 10–50 × 10^6^ cells/ml) was considerable on improving the cellular and structural functions of the bioprinted muscle tissue constructs, which significantly influenced in the tissue development and dimensional stability. To optimize the initial cell density in the bioprinted muscle constructs, the constructs with different cell densities were subcutaneously implanted in mice, providing a non-myogenic environment. The histological analyses indicate that the spatial organization of the bioprinted muscle constructs was maintained following implantation. Moreover, there was an increasing trend in the volume maintenance (implant’s thickness) and myofiber formation with increasing cell density; however, there were no significant differences in the dimensional maintenance and myofiber formation between the cell densities of 30 × 10^6^ and 50 × 10^6^ cells/ml. Cell densities higher than 30 × 10^6^ cells/ml may increase oxygen consumption by cells, while diffusion of oxygen decreases in denser tissues. The cell survival and tissue formation capability reach a plateau. Therefore, the initial cell density of 30 × 10^6^ cells/ml is optimal for the bioprinted muscle tissue constructs based on the myofiber formation and thickness maintenance after subcutaneous implantation.

The treatment of the muscle defect is a challenging clinical problem since a relatively large, thick muscle defect (>20% of the original muscle mass) suffers functional impartment and has no regenerative capacity^3^. Thus, the bioengineered muscle constructs should be examined using the muscle defect animal model that is more clinically relevant. Previously, various bioengineered skeletal muscle constructs were applied to muscle defected injury models, such as TA muscle defects in rats^29^, EDL muscle defects in mice^43^, quadriceps muscle defects in mice^44^, and vastus lateralis muscle defects in rabbits^45^; however, these approaches only achieved modest structural or functional recovery. A standardized rodent model of muscle defect injury created by excising ~20% of the middle of the TA muscle was reported^37^. This model allows functional assessment and shows no recovery of muscle weight and tetanic muscle force until 6 months after injury^37,46^. In this study, we used 30–40% excision of TA muscle in addition to ablation of EDL and EHL muscles to prevent compensatory hypertrophy^10^. Our modified TA defect model confirmed the irreversible structural and functional deficits with 25% and 54% reduction in muscle force and weight, respectively, following 2 months post-injury. For implantation, bioprinted muscle constructs were designed to be 10 × 7 × 3.6 mm^3^ in dimensions after 4–5 days differentiation in culture. The outcomes indicate the structural and functional skeletal muscle regeneration using the 3D biomimetic bioprinted muscle constructs, while the non-printed construct and cell-free construct (gel only) did not provide any therapeutic effects. The regenerated muscle tissue was highly mature and organized with vascular and neural integrity at 4 and 8 weeks after implantation. More importantly, the muscle force was recovered by up to 85% of normal muscle force at 8 weeks after implantation.

There are several advanced strategies to bioengineer skeletal muscle constructs with cellular orientation. For example, a skeletal muscle bundle-like structure with highly organized architecture was fabricated by polydimethylsiloxane (PDMS) molds^18^. This technique provided the structural maturation and force-generating capacity of neonatal rat myogenic cells in the construct. In another study, a scaffold-free skeletal muscle unit was fabricated by rolling the monolayer-cultured rat muscle cells into cylindrical forms, and this bioengineered muscle showed a therapeutic potential *in vivo*^29^. However, these strategies to bioengineer the skeletal muscles may not be clinically feasible. They are not appropriate to build clinically relevant sized muscle constructs that composed of multi-layered myofiber bundles. The bioengineered muscle constructs fabricated by the molding method had one layer of tri-bundle, and the rolling method showed a thin sheet form of the skeletal muscle unit. Moreover, these strategies were tested by the animal cell sources. We previously investigated a possibility of 3D bioprinting technology to produce human-scale tissue constructs^36^. In this study, we utilized 3D bioprinting strategy to build 3D skeletal muscle constructs using the clinically relevant human primary cells. We believe that this strategy can accelerate the clinical translation of the bioengineered skeletal muscles.

Although this study demonstrated the feasibility using 3D bioprinted muscle constructs containing human primary muscle cells, several challenges still remained for the future translation. Since we tested the bioprinting strategy for bioengineering skeletal muscle in the immunocompromised animal model, the host responses, including inflammatory response and foreign body reaction, in the regeneration process need to be investigated. It is also required to examine the systemic effects of the implanted cells for future translation. Thus, a study using a preclinical immune-competent animal model with an autologous cell source will be mandatory. In addition, accelerating functional integration of bioengineered skeletal muscle by host nerve is the most important factor for the clinical success. Adequate innervation of the implanted muscle constructs could prevent the severe muscular atrophy to achieve functional muscle recovery^47^. Therefore, neuronal components that facilitate the muscle cell survival, differentiation, and innervation can be incorporated to improve the muscle function. Potential introduction of neural cells, neurotrophins, or neurotransmitters can be advantageous to develop the accelerated muscle function recovery of the bioprinted muscle constructs^48–50^.

In summary, we designed and fabricated the implantable and biomimetic skeletal muscle construct based on the 3D bioprinting strategy for functional muscle regeneration. We examined that the bioprinted muscle constructs had the structural and cellular characteristics of the native muscle, which consisted of multi-layered myofiber bundles of highly viable, densely packed, and spatially organized hMPCs. We successfully implanted these bioprinted muscle constructs in the rat TA muscle defect. Highly organized muscle tissue regeneration with the vascularization and host nerve integrity resulted in the restoration of muscle function. Our 3D bioprinted skeletal muscles present a possibility for therapeutic effects to treat the muscle defect injuries and have implications for future translation.

## Methods

### Human muscle progenitor cell (hMPC) culture

Primary MPCs were taken from biopsies of human gracilis muscles (from a 51-year-old and a 64-year-old woman) as previously published^51^, following the Wake Forest University Institutional Review Board (IRB)-approved protocol. Briefly, the muscle biopsies (average 200 mg) were rinsed in PBS and minced to less than 1 × 1 mm^2^ following removal of tendon and fat tissues. The minced muscles were digested in DMEM (Gibco, Grand Island, NY) containing 0.2% (w/v) of collagenase type I (Worthington Biochemical, Lakewood, NJ) and 0.4% (w/v) dispase (Gibco) for 2 h at 37 °C. The digested muscles were washed with DMEM/F12 nutrient mix (1:1) (Gibco) consisting of 18% fetal bovine serum (FBS, Valley Biomedical Inc., Winchester, VA), 10 ng/ml human epidermal growth factor (hEGF), 1 ng/ml human basic fibroblast growth factor (hbFGF), 10 µg/ml human insulin, and 0.4 µg/ml dexamethasone (all from Sigma, St. Louis, MO). The muscle fibers were gently pipetted, filtered through a strainer with a pore size of 100 µm, and centrifuged at 1500 rpm for 5 min. The resulting pellet was resuspended in the medium. Single muscle fibers were plated into 35 mm dishes coated with collagen type I (1 mg/ml, BD, Clontech, Bedford, MA), and incubated overnight in the humidified atmospheric air including 5% CO_2_ at 37 °C. To remove contaminant cells such as fibroblasts, the supernatant containing non-adhered hMPCs was collected and transferred into collagen type I-coated 35 mm dishes. For primary cell culture, the medium was changed at 4 and 7 days, and the cells showed 80% confluence at 8–10 days. The cells were expanded in a growth medium composed of DMEM/high glucose, 20% FBS, 2% chicken embryo extract (Gemini Bio-Products, West Sacramento, CA), and 1% penicillin/streptomycin (Thermo Scientific Inc., Waltham, MA). Human MPCs were expanded up to passage 4 for bioprinting.

### Bioink preparation

In the printing process, three components were used to print the muscle constructs: the cell-laden bioink, the sacrificing acellular bioink, and the supporting PCL pillar. The cell-laden bioink composed of 20 mg/ml fibrinogen (Sigma), 35 mg/ml gelatin (Sigma), 3 mg/ml hyaluronic acid (HA, Sigma) and 10% (v/v) glycerol (Sigma) was prepared. Briefly, HA was dissolved in DMEM/high glucose at 37 °C with stirring overnight, and then glycerol was added to HA solution and stirred for 1 h. Fibrinogen and gelatin were added to the HA/glycerol solution by gentle shaking at 37 °C for 1 h. After dissolution, the solution was sterilized by a 0.45 µm syringe filter (Thermo Scientific). Lastly, the cells were mixed with the bioink by gentle pipetting. The sacrificing bioink was prepared by dissolving 35 mg/ml gelatin, 3 mg/ml HA, and 10% (v/v) glycerol in DMEM/high glucose, and then filtered. PCL (Mw; 43,000~50,000, Polysciences, Inc., Warrington, PA) was used for the polymeric pillar structure.

### Bioprinting process

All bioprinted muscle constructs were fabricated using our 3D ITOP system. This system is composed of an XYZ stage/controller, multiple dispensing modules, a pneumatic pressure controller, and a closed chamber with temperature controller and humidifier. The 3D skeletal muscle constructs with various dimensions were designed with 3D computer-aided design (CAD) modeling using our customized software. The CAD models were converted to a motion program, path information of each material was generated, and scanning speed, air pressure, and dispensing materials were controlled. The motion program was transferred to the operating computer of the 3D ITOP system.

To bioprint skeletal muscle constructs, all materials were aseptically inserted into different cartridges. The cell-laden bioink and sacrificial bioink were loaded into sterile plastic syringes at 37 °C and then cooled on ice for 10 min. The PCL polymer was loaded into a stainless-steel syringe which was heated at 95 °C for melting. The syringes were connected to microscale nozzles (300 µm in diameter, TECDIA, Inc., Tokyo, Japan) and inserted into the dispensing module, which was connected to the air pressure controller. The temperature of the closed aseptic chamber was maintained at 18 °C during the printing process. Before printing, the nozzles were aligned to determine their respective X-Y-Z offsets with minimum 0.01 mm accuracy. The cell-laden bioink was printed through a Teflon^®^ nozzle at a speed of 90 mm/min and air pressures ranging from 50 to 70 kPa. The gelatin sacrificial bioink was printed through a Teflon^®^ nozzle at a speed of 160 mm/min at 50–80 kPa. Dispensing speed and pressure of the PCL polymer through a metal nozzle was 75 mm/min and 780 kPa, respectively. After printing the constructs, 20 UI/ml of thrombin solution (Sigma) were treated on the printed constructs to crosslink the fibrinogen at room temperature for 30 min to 1 h, depending on the size of the constructs. The bioprinted constructs were cultured in the growth medium supplemented with aprotinin (20 µg/ml, Sigma). To induce the myofiber formation, the bioprinted muscle constructs were cultured in the differentiation medium composed of DMEM/high glucose, 2% horse serum (Gibco), 1% ITS (Lonza, Basel, Switzerland), 250 nM dexamethasone, 1% penicillin/streptomycin, and 20 µg/ml aprotinin. The medium was changed every other day.

### *In vitro* biological evaluations

Viability and differentiation of hMPCs in the printed and non-printed constructs were evaluated *in vitro*. For comparison, the bioprinted and non-printed (hMPCs in hydrogel without printing) muscle constructs were prepared with the same cell density (30 × 10^6^ cells/ml) and dimension (10 × 10 × 3 mm^3^). For optimizing cell density, the bioprinted muscles with various cell densities (10, 20, 30, and 50 × 10^6^ cells/ml) were fabricated. The viability of cells within the constructs was examined using a live/dead assay/cytotoxicity kit (L-3224; Life Technologies, Carlsbad, CA) and TUNEL assays (Trevigen, Gaithersburg, MD) following the manufacturer’s instructions. For the live/dead assay, the bioprinted muscle constructs were collected at 1, 6, or 9 days in culture. They were washed with PBS and incubated in the assay solution (50 µl/ml Cal-AM and 2 µl/ml Et-D in DMEM/high glucose) at room temperature for 40–60 min. After gently washing the constructs with PBS, 4 to 6 images of stained samples were taken using a confocal microscope (Leica TCS LSI Macro Confocal; Leica, Microsystems, Wetzlar, Germany). Using these images, the number of live cells (green) and dead cells (red) were manually counted, and cell viability (%) was quantified as the ratio of live cells to the total cells. To investigate the viability of cells inside of the constructs, a TUNEL assay was performed at 6 days in culture. Sections were prepared by fixing the bioprinted muscle constructs with 4% paraformaldehyde for 1 h and then cryosectioning with 6 µm thickness. With light microscope images (DM4000, Leica Microsystems) apoptotic cells (%) were calculated as the ratio of TUNEL^+^ cells (brown) to total cells (green) in a blinded fashion (*n* = 3).

To evaluate the differentiation of hMPCs in the bioprinted constructs and non-printed constructs *in vitro*, immunofluorescent staining for MHC and α-SA/laminin was performed. The constructs were fixed with 4% paraformaldehyde for 30 min and permeabilized in methanol at −20 °C for 20 min. For α-SA/laminin staining, the constructs were permeabilized in 0.1% Triton X-100 for 20 min and blocked using a serum-free blocking agent (X090930-1; Dako, Carpentaria, CA) at room temperature for 1 h. The samples were incubated with mouse anti-MF-20 antibody (MF20; 1 µg/ml; Developmental Studies Hybridoma Bank, Iowa City, IA), mouse anti-α-SA (a7811; 1:200 dilution; Sigma), and rabbit anti-laminin (ab11575; 1:300 dilution; Abcam, Cambridge, MA) antibodies for 1 h. The samples were then incubated with the secondary antibodies, Texas Red-conjugated anti-mouse (TI-2000; 1:200 dilution; Vector Labs, Burlingame, CA) or Alexa 488-conjugated anti-rabbit IgG (A11070; 1:200 dilution; Invitrogen, Eugene, OR), for 1 h. All antibodies were diluted with antibody diluent (S302283-1; Dako). The samples were mounted with VECTASHEILD Mounting Medium (H-1000; Vector Labs) or Prolong® Gold Antifade Mountant (P36930; Life Technologies) following incubation in DAPI (D1306; 1:1000 dilution; Life Technologies) for 10 min. The stained images were taken using a confocal microscope (FV10i; Olympus, Tokyo, Japan). For quantification of myofiber formation in the constructs, the area of MHC^+^ myofibers (mm^2^/mm^2^) was measured by using immunofluorescent staining images for MF-20 (×600) in a blinded fashion (*n* = 3, 4–7 random images per each sample). The α-SA^+^ area (%) and laminin^+^ area (%) of the non-printed and printed constructs were analyzed by immunofluorescent staining images for α-SA/laminin (×1200, *n* = 3).

### *In vivo* evaluations of bioprinted muscle construct for optimal cell density

All animal procedures were performed in accordance with a protocol approved by the Institutional Animal Care and Use Committee (IACUC) at Wake Forest School of Medicine. Male athymic mice (6–8 weeks old) were obtained from Charles River Laboratory (Wilmington, MA). Anesthesia was induced by using 3% isoflurane before surgical procedures, and bioprinted muscle constructs were ectopically implanted in the subcutaneous pockets created by dorsal paramedian longitudinal incisions, and skin was closed using non-absorbable sutures. For implantation, the bioprinted muscle constructs with cell densities of 10, 20, 30, and 50 × 10^6^ cells/ml (10 × 10 × 3 mm^3^ in dimension) were cultured in the growth medium for 1 day and then in the differentiation medium for another 3 days. Animals were euthanized 1, 2, and 4 weeks after implantation for histologic and immunohistologic analyses (48 nude mice, *n* = 4 in each group).

### A rat TA muscle defect model

All animal procedures were performed in accordance with a protocol approved by the IACUC at Wake Forest School of Medicine. The muscle defect model was created in RNU rats (male, 10–12 weeks old, Charles River Laboratory). Under anesthesia, the rats received a long incision on the skin of the left lower leg, and the fascia was separated from the muscles. EDL and EHL muscles were removed to rule out compensatory hypertrophy during muscle regeneration, and approximately 30–40% of the middle third of TA muscle was excised and weighed. TA muscle weight of each animal was estimated by the following equation: y (g) = 0.0017 × body weight (g)–0.0716^37^. The constructs were implanted in the excised sites and covered with fascia. Fascia and skin were closed using sutures or surgical staples. In this study, 5 groups were investigated, and each group had 4-week and 8-week time points (total 30 rats, *n* = 3); (1) sham (without defect), (2) non-treated (defect only), (3) gel only, (4) non-printed (cells in gel), and (5) bioprinted muscle construct. For implantation, the bioprinted muscle constructs were cultured in the growth medium for 1 day and then differentiated for 4–5 days. Bioprinted muscle constructs (30 × 10^6^ cells/ml) were fabricated with dimensions of 10 × 7 × 3 mm^3^. For non-printed construct, equal volumes of cell-laden composite hydrogel (30 × 10^6^ cells/ml) and gelatin sacrificing hydrogel were mixed by gentle pipetting. Groups of gel only, non-printed, and bioprinted constructs had the same dimension of the constructs for implantation.

### *In vivo* functional examinations

To evaluate restoration of muscle function, the tetanic force of TA muscle was measured with a dual-mode muscle lever system (Aurora Scientific, Inc., Model 305b, Aurora, Ontario, Canada) at 4 and 8 weeks after implantation in a blinded fashion (*n* = 3)^52,53^. Rats were anesthetized, and their body temperature was maintained at 37 °C. The left foot was attached to a food plate, and the knee and ankle were stabilized at 90° angle. Two sterilized platinum needle electrodes were placed in the posterior compartment of the lower leg along either side of the peroneal nerve, and the nerve was stimulated using a Grass stimulator (S88) at 100 Hz and 10 V with a pulse-width of 0.1 msec. Muscle force (N·mm/Kg) was calculated by (peak isometric torque × foot length)/body weight. After the functional assessment, TA muscles of each lower leg were collected and weighed. The percentage of TA muscle weight (% of contralateral) was calculated by the ratio of the weight of injured TA muscle to that of contralateral TA muscle (*n* = 3).

### Histologic and immunofluorescent analyses

For histologic evaluations of *in vivo* samples, the harvested tissues were freshly frozen in liquid nitrogen and cryosectioned into 6-µm-thick slices. Sectioned tissue slides were fixed with 4% paraformaldehyde for 10 min and then stained with H&E and Masson’s trichrome stain. The thickness of subcutaneous implants was measured using with H&E-stained longitudinal sections in a blinded fashion (*n* = 4 per each group, 3 random regions per each sample).

For immunofluorescent staining, the paraformaldehyde-fixed tissue sections were permeabilized in methanol at −20 °C and blocked using a serum-free blocking agent for 1 h at room temperature. The blocked sections were incubated with primary antibodies at room temperature for 1 h or incubated at 4 °C overnight. The slides were treated with secondary antibodies at room temperature for 40 min. Tissue sections were then mounted with VECTASHIELD Mounting Medium with DAPI and analyzed by fluorescent imaging using an upright microscope (Leica). Mouse on Mouse (M.O.M.) Kit (BMK-2202; Vector Labs) was used for reducing endogenous mouse antibody staining in any mouse-on-mouse application.

To examine the muscle regeneration of implanted bioprinted muscle constructs, tissue sections were double-stained with mouse anti-MF20 and rabbit anti-HLA-A (ab52922; 1:100 dilution; Abcam). MHC^+^ myofibers (/mm^2^) were counted, and area of MHC^+^ myofibers (mm^2^/mm^2^) was measured with immunofluorescent images for MHC (×600) or MHC/HLA (×600) staining in a blinded fashion (*n* = 4, 3–4 random fields in each sample). In addition, we counted the number of HLA^+^ myofibers (/mm^2^) and HLA^+^ myofibers (%) and measured the area of HLA^+^ myofibers (mm^2^/mm^2^) to evaluate muscle differentiation of implanted hMPCs. Also, co-localized HLA^+^/MHC^+^ cells (% of HLA^+^/MHC^+^ cells per MHC^+^ cells) were evaluated (*n* = 3). The diameter of MHC^+^/HLA^+^ cells (µm) (*n* = 3, 3 random fields per sample) were measured with immunostaining with MHC/HLA of cross-sectional samples. All images were analyzed with Image J software (National Institutes of Health, Bethesda, MD).

For vascularization, tissue sections were stained with rabbit anti-vWF (A0082; 1:400 dilution; Dako) and mouse anti-α-SMA (sc-32251; 1:50 dilution; Santa Cruz Biotechnology, Santa Cruz, CA). Neuromuscular junctions were visualized by immunostaining with rabbit anti-NF (N4142; NF200, 1:80 dilution; Sigma)/α-Btx (B13423; Alexa Fluor 594 conjugate; 1:100 dilution; Invitrogen), chicken anti-NF (ab4680; 1:1000 dilution; Abcam)/rat anti-AChR (ab24719; 1:100 dilution; Abcam)/mouse anti-MF-20 or chicken anti-NF/rat anti-AChR/rabbit anti-HLA A. Secondary antibodies such as Alexa 488-conjugated anti-rabbit or anti-chicken antibody (A11070; A11039; 1:200 dilution; Invitrogen), Texas Red-conjugated anti-mouse, anti-rabbit, or anti-rat antibody (TI-2000; TI-1000; TI-9400; 1:200 dilution; Vector Labs), or Cy5-conjugated anti-mouse or anti-rabbit antibody (A10524; A10523; 1:200 dilution; Invitrogen) were used. For quantification of vascularization, vessels/field and area of vessels/field (µm^2^) were measured with immunofluorescent images for vWF/α-SMA (*n* = 3). NMJ/field were counted with immunofluorescent images for MHC/AChR/NF (×400) (*n* = 3 per group, 3 random fields per sample).

### Statistical analysis

Results were analyzed with Origin Pro 8.5 (OriginLab Co, Northampton, MA) and SPSS software (SPSS, version 19; IBM, Armonk, NY). One-way analysis of variance (ANOVA), Tukey *post hoc* testing and Student *t*-test were applied to mean comparisons. Variables are expressed as a mean ± standard deviation, and differences between experimental groups were considered statistically significant at *P* < 0.05.

## Electronic supplementary material

Supplementary Information
