# Supplementary material for: 3D Bioprinted Human Skeletal Muscle Constructs for Muscle Function Restoration
Source: Sci Rep. 2018 Aug 17;8:12307. doi: 10.1038/s41598-018-29968-5 (PMC6098064; doi:10.1038/s41598-018-29968-5)
Supplement: Supplementary file 1 — Supplementary Information [file 41598_2018_29968_MOESM1_ESM.docx]

**Supplementary Information**

**3D Bioprinted Human Skeletal Muscle Constructs for Muscle Function Restoration**

**Ji Hyun Kim^1^, Young-Joon Seol^1^, In Kap Ko^1^, Hyun-Wook Kang^1^, Young Koo Lee^1,3^, James J. Yoo^1,2^, Anthony Atala^1,2^, and Sang Jin Lee^1,2,^***

^1^Wake Forest Institute for Regenerative Medicine, Wake Forest School of Medicine, Winston-Salem, NC 27157, United States

^2^School of Biomedical Engineering and Sciences, Wake Forest University-Virginia Tech, Winston-Salem, NC 27157, United States

^3^Department of Orthopedic Surgery, Soonchunhyang University Bucheon Hospital, Bucheon, Gyeonggi-Do 420-726, Republic of Korea

*sjlee@wakehealth.edu

**
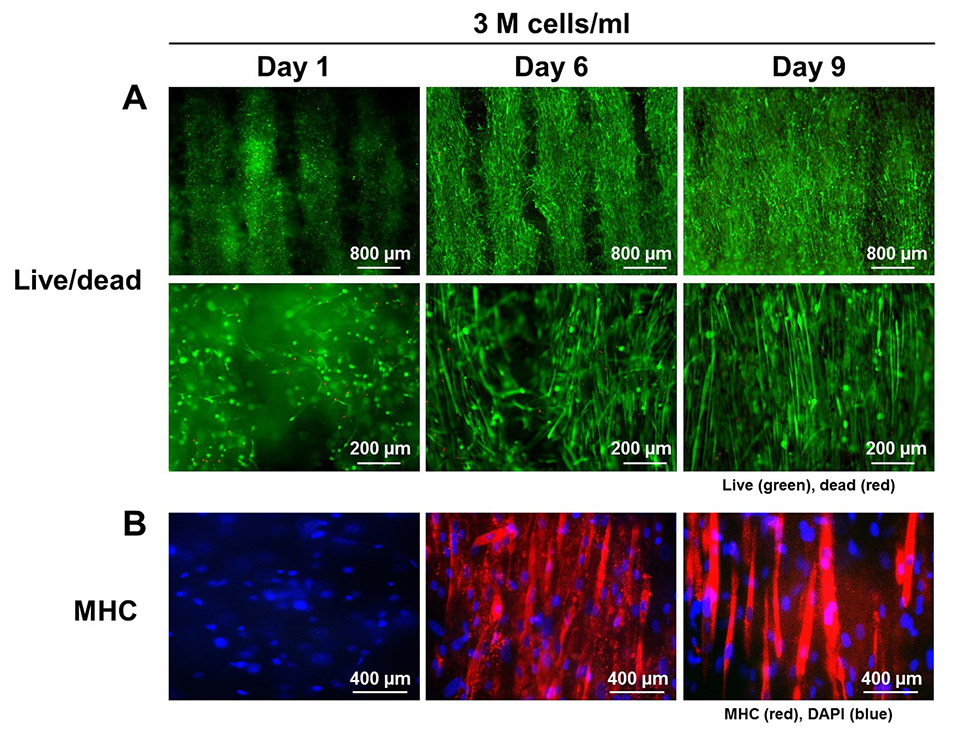
**

**Fig. S1.** Cell viability and differentiation of bioprinted muscle constructs. (A) Live/dead staining images of bioprinted muscle constructs with 3 × 10^6^ cells/ml at 1, 6, and 9 days in culture (top: low magnification, bottom: high magnification). The live/dead staining indicates high cell viability (green: live cells, red: dead cells). The hMPCs with PCL pillar showed unidirectionally organized cellular morphologies that are gradually aligned along the longitudinal axis of bioprinted construct. (B) MHC immunostaining showed the differentiation of hMPCs and their alignment in bioprinted muscle construct.

**
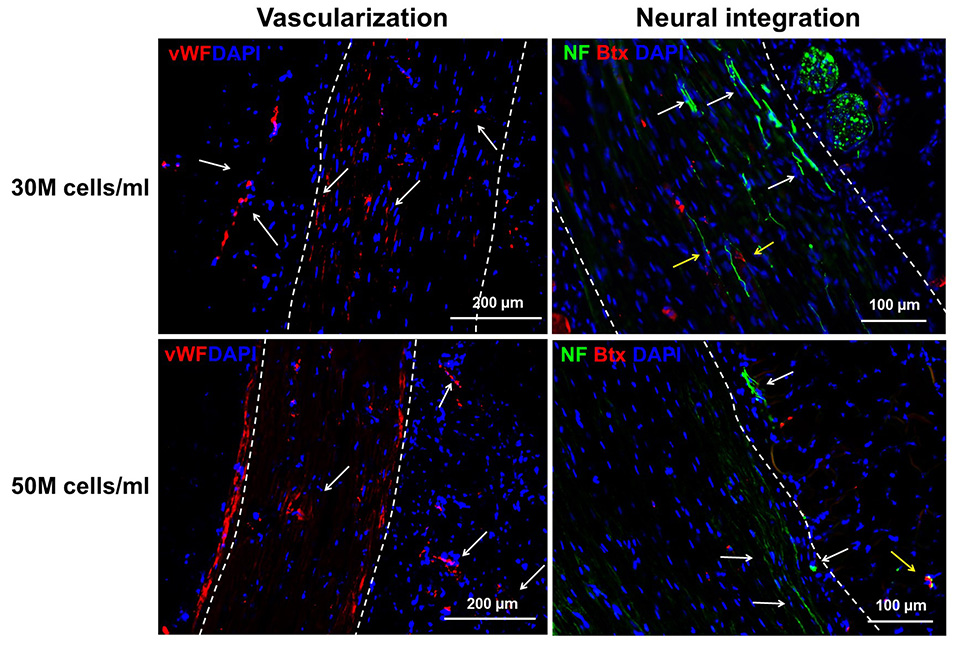
**

**Fig. S2.** Vascularization and neural integration of the ectopically implanted bioprinted muscle construct. Immunofluorescence of vWF (red) and NF (green)/α-Btx (Red) indicate vascular (white arrow, vWF+) and neural integration (white arrow, NF+ nerve; yellow arrow, NF+/α-Btx+ neuromuscular junction) in bioprinted muscle construct with cell densities of 30 × 10^6^ cells/ml and 50 × 10^6^ cells/ml after 2 weeks of implantation.

**
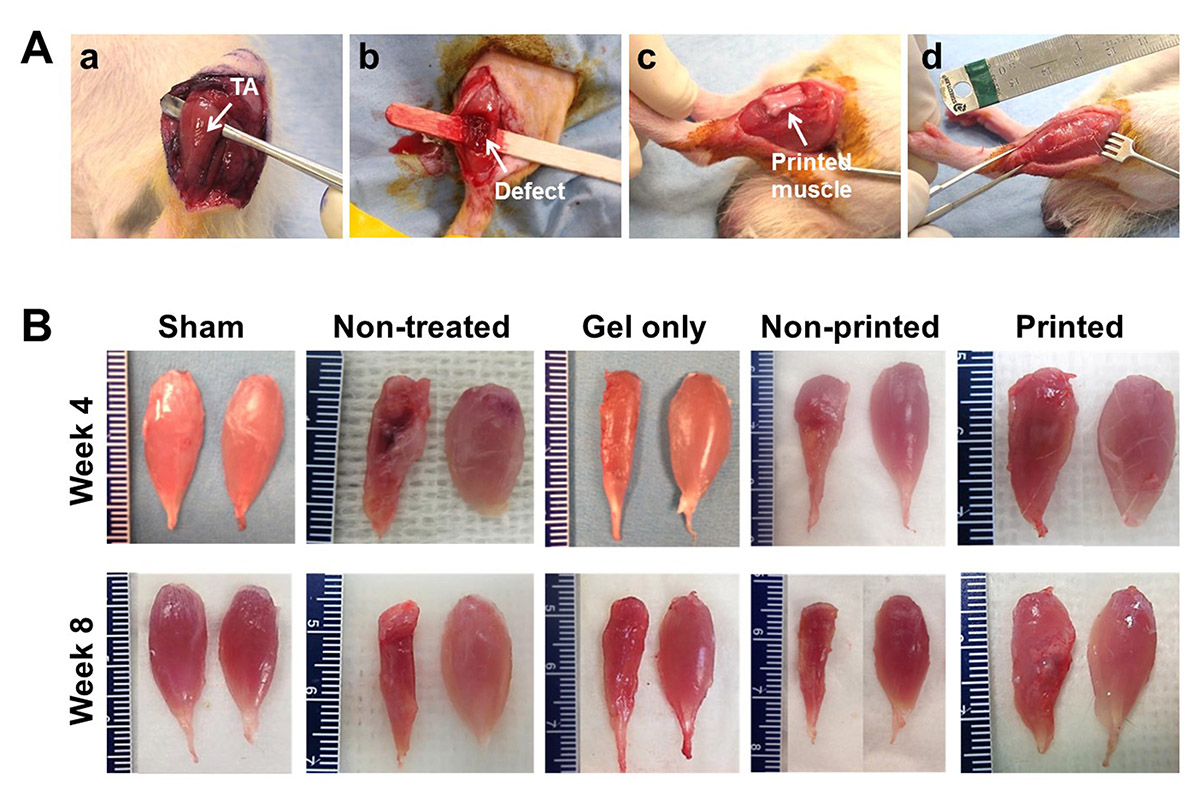
**

**Fig. S3.** Rat VML injured model. (A) Surgical procedure of volumetric TA muscle defect and implantation of bioprinted muscle construct; (a) exposure of TA muscle, (b) creation of the VML injury by removing 30 – 40% of TA muscle after ablation of EDL and EHL muscles, (c) implantation of bioprinted construct, and (d) closure using fascia. (B) Gross appearance of TA muscles of left legs (left, defected TA) and right legs (right, contralateral normal TA) at 4 and 8 weeks after implantation.

**
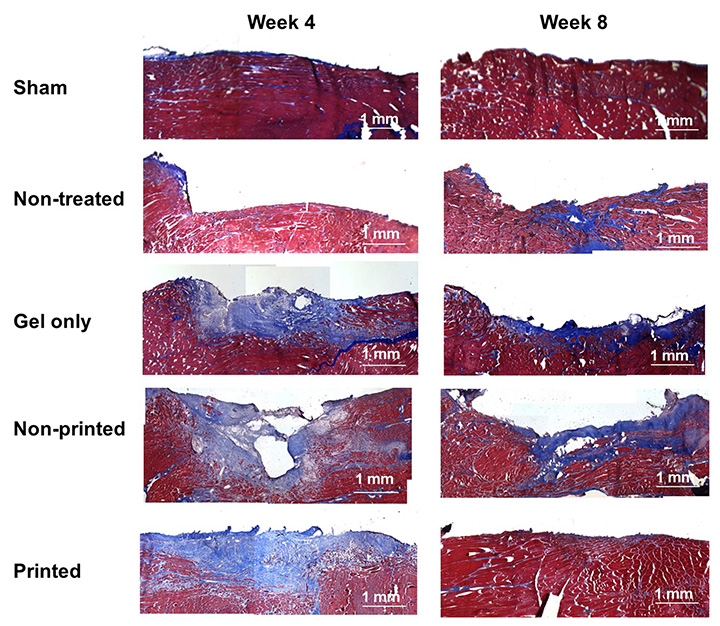
**

**Fig. S4.** Masson’s trichrome staining images at 4 and 8 weeks of implantation.

**
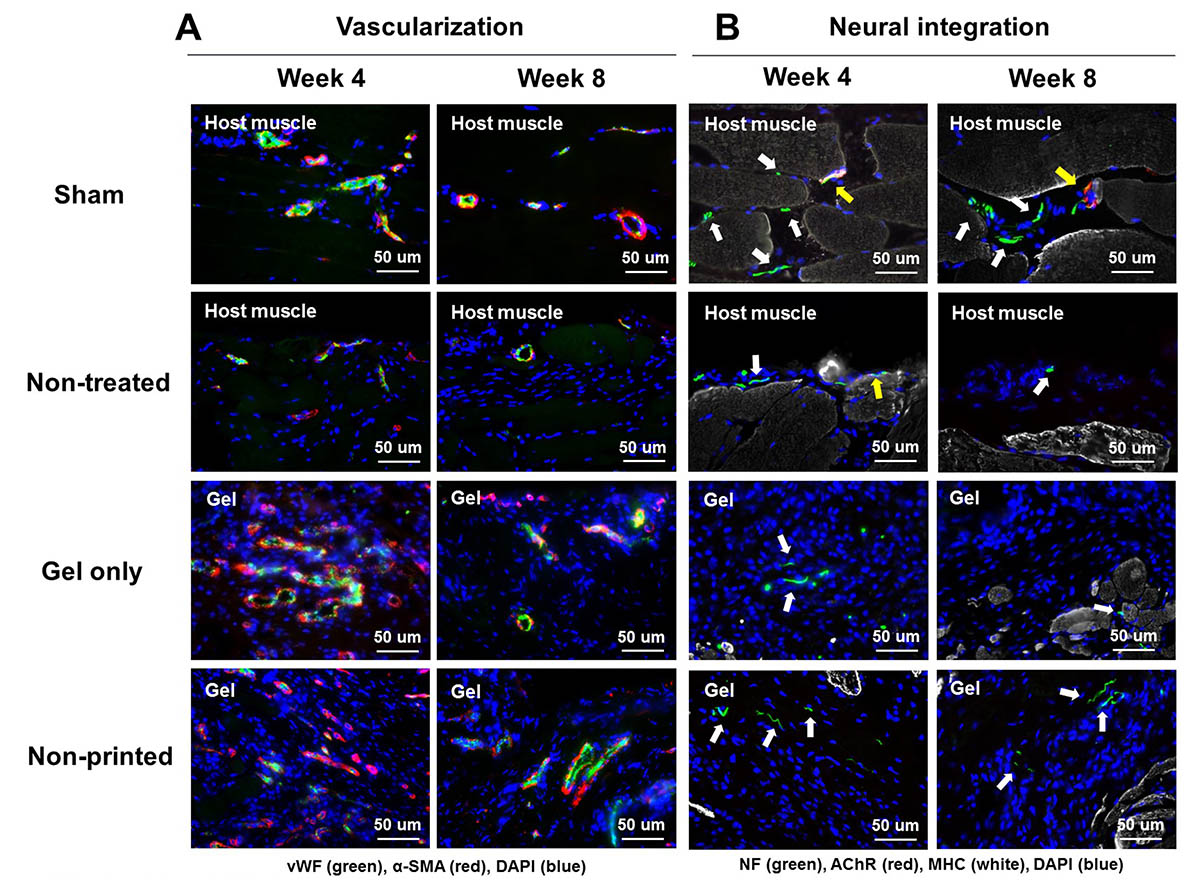
**

**Fig. S5.** Immunofluorescence of vascularization and neural integration in the implanted region at 4 and 8 weeks after implantation. Immunofluorescent images of (A) vWF (green)/α-SMA (red) and (B) NF (green)/AChR (red)/MHC (white). White arrow indicates a NF+ neuron and yellow arrow indicates a NF+/AChR+/MHC+ neuromuscular junction.
